# Knowledge of Signs and Symptoms of Heart Attack and Stroke among Singapore Residents

**DOI:** 10.1155/2014/572425

**Published:** 2014-04-10

**Authors:** Joy Li Juan Quah, Susan Yap, Si Oon Cheah, Yih Yng Ng, E. Shaun Goh, Nausheen Doctor, Benjamin Sieu-Hon Leong, Ling Tiah, Michael Yih Chong Chia, Marcus Eng Hock Ong

**Affiliations:** ^1^Yong Loo Lin School of Medicine, National University of Singapore, 10 Medical Drive, Singapore 117597; ^2^Department of Emergency Medicine, Singapore General Hospital, Outram Road, Singapore 169608; ^3^Medical Department, Singapore Civil Defence Force, 91 Ubi Avenue 4, Singapore 408827; ^4^Acute and Emergency Care Centre, Khoo Teck Puat Hospital, 90 Yishun Central, Singapore 768828; ^5^Emergency Medicine Department, National University Hospital, 5 Lower Kent Ridge Rd, Singapore 119074; ^6^Accident & Emergency Department, Changi General Hospital, 2 Simei Street 3, Singapore 529889; ^7^Emergency Department, Tan Tock Seng Hospital, 11 Jalan Tan Tock Seng, Singapore 308433; ^8^Office of Clinical Sciences, Duke-NUS Graduate Medical School, 8 College Road, Singapore 169857

## Abstract

*Aim*. To determine the level of knowledge of signs and symptoms of heart attack and stroke in Singapore resident population, in comparison to the global community. * Methods*. A population based, random sample of 7,840 household addresses was selected from a validated national sampling frame. Each participant was asked eight questions on signs and symptoms of heart attack and 10 questions on stroke. * Results. *The response rate was 65.2% with 4,192 respondents. The level of knowledge for preselected, common signs and symptoms of heart attack and stroke was 57.8% and 57.1%, respectively. The respondents scored a mean of 5.0 (SD 2.4) out of 8 for heart attack, while they scored a mean of 6.8 (SD 2.9) out of 10 for stroke. Respondents who were ≥50 years, with lower educational level, and unemployed/retired had the least knowledge about both conditions. The level of knowledge of signs and symptoms of heart attack and stroke in Singapore is comparable to USA and Canada. * Conclusion*. We found a comparable knowledge of stroke and heart attack signs and symptoms in the community to countries within the same economic, educational, and healthcare strata. However older persons, those with lower educational level and those who are unemployed/retired, require more public health education efforts.

## 1. Introduction

Heart attack and stroke are leading causes of death globally. The World Health Organisation estimates that 7.3 million deaths globally were due to coronary heart disease and 6.2 million were due to stroke in 2008 [1]. By 2030, almost 23.6 million people will die from cardiovascular disease every year [2].

Singapore has gone through rapid economic progression since the postwar era. In 2010, she was ranked third in the world by the International Monetary Fund organization in terms of gross domestic product (GDP) per capita [3]. According to United Nations statistics, from 2005 to 2010, Singapore is ranked the 1st globally for lowest infant mortality rate at 1.92 infant deaths per 1 000 live births and ranked 10th for life expectancy at birth with a mean of 80.6 years in 2010 [4]. In terms of literacy rate, Singapore has been ranked by the World Bank to be within the top 30 nations in the world, with a literacy rate of 96% [5].

The paradigm of public health care in Singapore has shifted from managing infectious diseases to “lifestyle diseases,” including tackling the increasing incidence of strokes and heart attacks. It has been projected that, between 2006 and 2015, the number of acute resident hospitalisations for ischaemic heart disease and stroke would have increased by 21% and 57% each year, respectively [6]. In 2011, ischemic heart disease accounted for 16.4% of all principle causes of death while cerebrovascular disease (including stroke) accounts for 9.0%, being the 2nd and 4th most common causes of death, respectively [7]. This is similar to United States, where heart attack is the most common cause of death and stroke is the 4th [8] and similarly in Europe [9].

Both heart attack and stroke have better outcomes with time-sensitive treatments such as percutaneous coronary intervention (PCI) and thrombolysis [10–12]. This makes it important for prompt recognition of signs and symptoms of heart attack and stroke, allowing earlier presentation to the hospital for immediate treatment, reducing mortality and morbidity.

For heart attack symptoms, studies conducted in the United States [13], United Kingdom [14], and Australia [15], showed a median interval of 2.2 hours to 6.4 hours before presentation. Our sole local study on presentation times found that the median time from the first onset of chest pain to presentation at the Emergency Department (ED) for ST elevation myocardial infarction is rather similar, with a delay of 173 mins (interquartile range [IQR]: 270 mins), and median time from the worst chest pain to presentation at ED was at 131 mins (IQR: 191 mins) [16].

For stroke, the same dilemma applies for delayed time presentation from symptom onset to hospital. Hospitals in the USA report that only 59% of stroke patients arrive in hospital within 3 hours of onset of symptoms [17]. Data from Europe [18] shows 40% to 56% arrive within 6 hours. A Singapore study performed in 1997 shows that similarly 41.4% arrive within 3 hours, 54.5% within 6 hours, and 68.5% within 12 hours [19]. Inability to recognize signs and symptoms of acute stroke has been cited as an important reason for delayed presentation.

In Singapore, there has been no study conducted on community understanding of heart attack symptoms. For stroke symptoms, there was only a small study of 150 stroke patients performed in 1997, evaluating their knowledge [20].

This study aimed to determine the current level of knowledge of the signs and symptoms of heart attack and stroke in the Singapore resident population, in comparison to other countries in the world with similar economic, educational, and health-care standards. We hypothesize that, given the similar time delays from the first presentation of symptoms to ED attendance, the level of knowledge for both conditions, compared to the rest of the developed world, may not differ much.

## 2. Methods

Singapore is a country with a land area of 712.4 square kilometers and a population of 4.98 million in 2009 [21]. The population is multiracial with the major ethnic groups being Chinese, Malay, and Indian.

The Health Promotion Board conducts an annual population-based study known as the Omnibus Survey to assess the current level of health knowledge and to gauge the efficacy of its various health programmes. In 2009, 7,840 household addresses were randomly selected using a 2-stage stratified sampling design. This study was exempted from ethics committee review as it was an anonymous survey.

Trained interviewers visited each selected household, from November 2009 to March 2010, to conduct face-to-face interview in one of the four national languages: English, Mandarin, Malay, or Tamil. From each household, the “Next Birthday” method was employed to select either a Singapore citizen or permanent resident aged between 18 and 69 to participate. Those not contactable after 3 visits at 3 different timings or refused to be interviewed or do not speak any of the four national languages were considered as nonrespondents.

Each respondent was given eight questions on signs and symptoms of heart attack and ten questions on stroke, in a True/False manner. For example, respondents would be given a statement, “Prolonged crushing, squeezing, or burning pain in the centre of the chest is a sign/symptom of heart attack” (True/False). Each question is scored 0 for a wrong answer and 1 for the correct answer, with a minimum score of 0 and a maximum score of 8 marks or 10 marks, respectively, for heart attack and stroke. These scores were then summed up and categorized as either low or high scores according to the following scale: for heart attack: low 0–4 points (≤50%) and high 5–8 points (>50%), while, for stroke: low 0–6 points (≤50%) and high 7–10 points (>50%). Although these scales, like most, are arbitrary, they serve the purpose of allowing for the standardized comparison of knowledge levels among groups.

The signs and symptoms of both heart attack and stroke were obtained from the Singapore Health Promotion Board Public Health Educational Resources [22, 23], to assess the effectiveness of current health education campaigns addressing these items.

### 2.1. Signs and Symptoms of Heart Attack

Consider the following:

1. prolonged crushing, squeezing, or burning pain in the center of the chest,
2. pain that radiates from the chest area to the neck, arms, shoulders, or the jaw,
3. shortness of breath,
4. dizziness,
5. nausea,
6. chills and sweating,
7. weak pulse,
8. cold and clammy skin, gray pallor, a severe appearance of illness.

### 2.2. Signs and Symptoms of Stroke

Consider the following:

1. sudden numbness or weakness usually on one side of the body,
2. sudden confusion or a fit,
3. difficulty in speaking or understanding,
4. sudden difficulty in seeing in one or both eyes,
5. sudden difficulty in walking,
6. difficulty in swallowing,
7. sudden severe headache with no known cause,
8. loss of concentration and memory,
9. loss of control of passing urine or passing motion (incontinence),
10. sudden severe giddiness, loss of balance, or coordination.

All data were analysed using the statistical package SPSS (version 17.0; SPSS Inc., Chicago, IL). Total counts of each symptom correctly identified were tabulated and overall percentages of correct answers were calculated. The heart attack and stroke scores were summed up for each respondent, and the overall results of the respondents were described as mean scores with a calculated standard deviation. The *P* values with 95% confidence intervals were analysed by an unpaired two-tailed* t-*test, stratifying data by the studied variables, such as age and gender. Statistical significance was set at *P* < 0.05.

## 3. Results

Of the 7,840 households randomly selected from the validated sampling frame, a total of 4,192 respondents participated in the survey, giving a response rate of 65.2%. The sociodemographics of the respondents are shown in Table 1, which is similar to the Singapore resident population, corroborated with the population census in 2009.

In this study, the current level of knowledge for both signs and symptoms of heart attack and stroke was fair regarding both conditions, in almost equal proportions, 57.8% and 57.1%, respectively. The respondents scored an overall mean of 5.0 (SD = 2.4) out of 8 for heart attack, while they scored an overall mean of 6.8 (SD = 2.9) out of 10 for stroke (Table 2).

85.1% of the respondents correctly identified prolonged crushing, squeezing, or burning pain in center of heart as a symptom of heart attack, while 72.9% recognized shortness of breath as another symptom. Only 66.6% correctly identified pain radiating from chest area to neck, arms, shoulders, or jaw as a possible presenting symptom.

**Figure 1:**
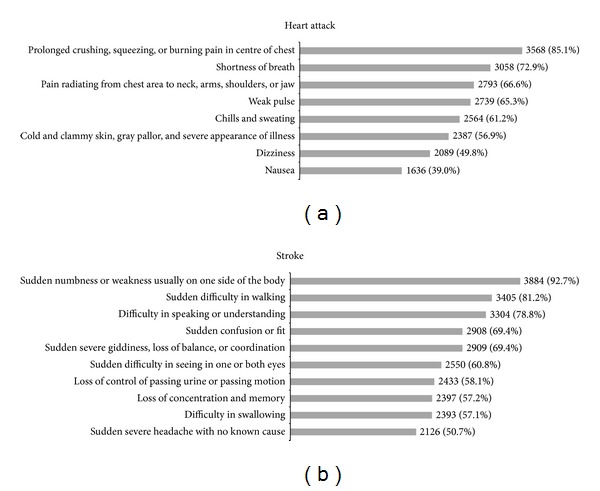
Proportion of respondents who were aware of the signs and symptoms of heart attack and stroke (*N* = 4,192).

Regarding signs and symptoms of stroke, 92.7% of the respondents were able to recognize sudden numbness or weakness usually on one side of the body as a symptom, and 81.2% correctly identified sudden difficulty in walking while 78.8% agreed that difficulty in speaking or understanding speech can also be a symptom (Figure 1).

Bivariate analysis of the independent variables stratified by high/low heart attack and stroke knowledge scores was conducted and the results are shown in Table 3.

Respondents ≥50 years old had the least knowledge for both conditions, 53.2% for heart attack knowledge compared to 58.9% for those aged between 36 and 49, and 61% for those aged ≤35 (*P* < 0.001). Similarly, the stroke knowledge was 52.4% for those aged ≥50 compared to 60.2% for those aged 36–49 and 58.4% for those aged ≤35 (*P* < 0.001).

Respondents with a lower educational level exhibited slightly lower scores for both conditions. Those whose lowest educational level was the Primary School Leaving Examination or GCE “O-” Level or GCE “N-” Level certification scored 53.4% for the heart attack knowledge, as compared to 62.8% and 64.3% for those with a GCE “A-” Level/Diploma or a University Degree, respectively (*P* < 0.001). The same was observed for their stroke knowledge of 53.9%, compared to 60.8% and 62.2% for those with a GCE “A-” Level/Diploma or a University Degree, respectively (*P* < 0.001).

Respondents who were unemployed/retired at time of interview exhibited lower scores for both conditions. For heart attack knowledge, they scored 52.6% as compared to 60.8% for those who were employed (*P* < 0.001). For stroke knowledge, they scored 52.3% as compared to 59.8% for those who were employed (*P* < 0.001).

Multiple logistic regression model was performed using scores on the heart attack and stroke knowledge questions as the dependent variable. The independent variables entered into the model were age, gender, race, education level, and occupational status. The results showed that those with higher knowledge for both disease conditions were more likely to be female, to have higher levels of education, and were employed (Table 4).

## 4. Discussion

In this study, we found that the current level of knowledge for signs and symptoms of heart attack and stroke was fair in the resident population. Those aged ≥50, with lower educational level, unemployed, or retired were the least knowledgeable.

## 5. Heart Attack Knowledge

Delay from symptom onset in a heart attack to presentation at hospital is an international concern. As mentioned in the Introduction section, studies conducted in the United States [13], United Kingdom [14], and Australia [15] showed similar median interval, of 2.2 hours to 6.4 hours before presentation, compared with Singapore [16]. One study suggested the most significant contributor to delayed treatment is the patient's ability to recognize the signs and symptoms of a heart attack [24]. An acute heart attack victim is popularly described as experiencing sudden excruciating chest pain, clutching onto one's chest, and collapsing. Other symptoms such as dyspnea, nausea, or syncope are lesser known [14]. In a major study in the USA, one-third of 434,877 subjects with confirmed diagnosis of myocardial infarction did not have chest pain on presentation [25]. When chest pain is not the main presenting complaint, patients may be confused about the severity of their symptoms and thus postpone seeking treatment.

From a global viewpoint, the level of knowledge of signs and symptoms of heart attack in Singapore is comparable to USA and Canada [26, 27]. From 2005 to 2009, the Centers for Disease Control in USA collated data nationwide via telephone interviews with a total of 103,262,115 respondents on heart attack knowledge. Their aim was to compare knowledge between the nonrural and rural populations. Singapore is a city-state, thus singling out their nonrural population analysis for comparison; their results showed a higher knowledge, with 92.8% identifying at least one symptom correctly, compared to 85.1% in Singapore. They found the more educated and younger adults (19 to 65 years old) to have higher knowledge, congruent with our study. Unlike ours, there was also a racial and gender discrepancy with Hispanics and women scoring lower [26]. In Vancouver, Canada, an urban study published in 2008 showed more similar results to ours, with 83.6% identifying at least one out of 10 symptoms correctly. They also found that level of knowledge was higher in young respondents with higher education level and higher annual household income [27]. In Victoria, Australia, a similar study was also conducted in 2002, with similar results of 84.6% identifying at least one symptom correctly out of 10 symptoms. Respondents with higher educational levels reported higher number of symptoms correctly [28].

## 6. Stroke Knowledge

Regarding stroke knowledge, numerous studies have been done in USA, in different states. A large-scale study performed by the Centers for Disease Control, in 17 states, showed that, in 2001, public knowledge of major warning signs of stroke was high, with 94.1% of the 61 019 respondents being able to identify at least one stroke symptom, with the most commonly identified being sudden numbness or weakness of the face, arm, or leg [29]. This level of stroke knowledge appears to be comparable to our study of 92.7%. The study did not carry out stratification studies. Further analysis of 2 published articles in the USA, performed in similar urban adult populations, in Ohio, 2003, and Michigan, 2002, concurred with our study that younger respondents and those with higher educational level have a higher stroke knowledge level [30, 31]. Telescoping into the Asia Pacific Region, in Victoria, Australia, a similar published study in 2001 shows 85.5% of 822 respondents correctly listing at least one stroke symptom [32]. This appears to be comparatively lower as compared to USA and Singapore. Despite that difference, again, stroke knowledge was found to be higher in those who are more educated. Age differences were not studied in this paper.

## 7. Taking One Step Forward 

Overall, the heart attack and stroke knowledge in the Singapore resident population appears to be somewhat similar, in comparison to countries within the same economic, educational, and healthcare strata.

In USA, an initiative known as the Public Health Action Plan to Prevent Heart Disease and Stroke has been in place since 2003 [33]. A “Communications Implementation” group is tasked with effectively communicating the urgency and importance of preventing heart disease and stroke through a long-term strategy of public information and education. In their 2008 updated publication, their action steps included crafting clear, attention-grabbing public health messages with a social marketing strategy, to determine media avenues, understand the changing dynamics and interactive nature of web-based communications, employ “expression in popular humor,” and identify target audiences [34].

A similarly comprehensive plan is in place in Canada for both heart attack and stroke knowledge, known as the Canadian Heart Health Strategy and Action Plan, started in 2006 with its latest executive summary published in 2009 [35].

In Singapore, public health initiatives are primarily managed by government statutory boards, the Singapore Health Promotion Board (HPB). HPB concentrates on publishing informative and interactive content online, organizing nationwide campaigns complete with public health exhibitions, and putting up posters in public areas for the adult population. The Board also engages the student population during their health education curriculum classes, coupled with field trips to the HPB's health zone interactive exhibition periodically. Currently, we lack the supportive statistics to determine the effectiveness of such interventions. Also, our interventions are mainly targeted at the literate, working, and studying populations. This can explain the results of this paper, as to why older, unemployed/retired respondents with lower educational levels had a lower level of knowledge in cardiovascular signs/symptoms.

Even so, this study has shown that Singapore's community level understanding of sign/symptoms of heart attack and stroke is comparably similar to other developed countries with comprehensive action plans. This conclusion is heartening and assuring that public school health education and continuing public health advocacy by the Singapore Health Promotion Board appears to be heading in a positive direction.

At the same time, we recognize the need for greater public knowledge of signs and symptoms of heart attack and stroke in general. Furthermore, any public health education needs to emphasize the less common presenting complaints of both conditions. As with Canadian and American action plan experiences, continual reassessment of methods employed to promote public health messages is important to maintain relevance. In this manner, this study could be used as a baseline, to measure the effectiveness of future public health campaigns and to correlate with studies performed after intervention.

More targeted public health measures can be taken to raise knowledge in the appropriate groups, for example, the group ≥50 years old, which are at higher risks for both heart attack and stroke. Another group to consider is the unemployed and retired, as campaigns in workplaces may prove futile to aid the understanding of this group. Public health campaigns may need to be carried out in the media such as television and radio, with supporting talks and poster exhibitions in common public-use areas such as community centres, bus stops, or train stations. Addressing those with a lower educational level may require that the information in the campaigns be kept simple and succinct.

We intend to follow up with public health education efforts, which should be studied for their effectiveness in eventually lowering morbidity and mortality, as such, becoming a springboard for comparison before and after intervention.

## 8. Limitations

Two possible limitations of this study include nonrespondent and interviewer biases. As 34.8% of the selected households did not participate in the survey, the findings might be compromised by nonrespondent bias, although there is no reason to believe that nonresponders were more or less likely to be knowledgeable. To minimize interviewer bias, intensive training was given to all interviewers to standardize method of asking, prior to commencement of study. Interviewers were also randomly shadowed by staff of the Health Promotion Board to ensure quality of the interviews remained consistent. The strength of this study is the close relation of the demographics of the sample population to the true Singapore resident population in the survey year of 2009 [21].

This study has also made the assumption that being equipped with knowledge will translate into earlier treatment. However, some studies [36, 37] have illustrated that there may be other barriers to early presentation to the hospital. These include a victim's perception of severity of symptoms, the person present with the individual at onset of symptoms, first contact for help (e.g., family member and family doctor), financial concerns, insurance coverage, and even previous negative experiences with healthcare institutions. Locally, no similar study has been conducted. Further studies in our local setting to explore other possible reasons why heart attack and stroke patients are not presenting to hospital earlier may be helpful.

## 9. Conclusion

We found a comparable knowledge of stroke and heart attack signs and symptoms in the community, to countries within the same economic, educational, and healthcare strata. However, there are still pockets of community which require more public health education efforts.

## Figures and Tables

**Table 1:** Demographics of survey respondents (*N* = 4,192).

	Survey respondents aged 18–69 years *N* (%)
Age	
≤35	1381 (32.9)
36–49	1453 (34.7)
≥50	1358 (32.4)
Gender	
Female	2256 (53.8)
Male	1936 (46.2)
Race	
Chinese	2983 (71.2)
Malay	645 (15.4)
Indian	408 (9.7)
Others	156 (3.7)
Educational level	
PSLE^#^/“O-”/“N-” Levels	2307 (55.0)
“A-” Levels/Diploma	1169 (27.9)
Degree	690 (16.5)
Refused/do not know/not sure	26 (0.6)
Occupational status	
Employed	2658 (63.4)
Unemployed/retired	1534 (36.6)

^#^PSLE: Primary School Leaving Examination.

**Table 2:** Descriptive statistics of heart attack and stroke knowledge scores of survey respondents.

	Heart attack knowledge score	Stroke knowledge score
Score category^#^		
Low score	42.2%	42.9%
High score	57.8%	57.1%

Range of knowledge scores	0–8	0–10

Mean knowledge scores	5.0	6.8
Standard deviation	2.4	2.9

^#^Correct answers received one point and were calculated separately using the following scale.

For the heart attack knowledge scores: low scores = 0–4 points or ≤50% and high scores = 5–8 points or >50%. For the stroke knowledge scores: low scores = 0–6 points or ≤50% and high scores = 7–10 points or >50%.

**Table 3:** Bivariate analysis of knowledge of signs and symptoms of heart attack and stroke score by independent variables.

	Heart attack knowledge score		Stroke knowledge score	
	Low 0–4	High 5–8	Low 0–6	High 7–10
Age				
≤35	39.0%	61.0%	41.6%	58.4%
36–49	41.1%	58.9%	39.8%	60.2%
≥50	46.8%	53.2%	47.6%	52.4%
*P* value	*P* < 0.001		*P* < 0.001	
Gender				
Female	40.6%	59.4%	41.8%	58.2%
Male	44.1%	55.9%	44.3%	55.7%
*P* value	*P* < 0.05		Not significant	
Race				
Chinese	43.3%	56.7%	43.1%	56.9%
Malay	39.2%	60.8%	41.4%	58.6%
Indian	41.4%	58.6%	42.6%	57.4%
Others	35.3%	64.7%	46.8%	53.2%
*P* value	Not significant		Not significant	
Educational level				
PSLE^#^/“O-”/“N-” Levels	46.6%	53.4%	46.1%	53.9%
“A-” Levels/Diploma	37.2%	62.8%	39.2%	60.8%
Degree	35.7%	64.3%	37.8%	62.2%
*P* value	*P* < 0.001		*P* < 0.001	
Occupational status				
Employed	39.2%	60.8%	40.2%	59.8%
Unemployed/retired	47.4%	52.6%	47.7%	52.3%
*P* value	*P* < 0.001		*P* < 0.001	

^#^PSLE: Primary School Leaving Examination.

**Table 4:** Multiple logistic regression analysis of factors associated with high heart attack and stroke knowledge score.

	Heart attack knowledge score Adjusted OR^a^ (95% CI^b^)	Stroke knowledge score Adjusted OR^a^ (95% CI^b^)
Age (years)		
≤35	1.0	1.0
36–49	1.0 (0.8, 1.1)	1.1 (0.9, 1.3)
≥50	0.9 (0.8, 1.1)	0.9 (0.8, 1.1)
Gender		
Female	1.0	1.0
Male	0.8 (0.7, 0.9)*	0.8 (0.7, 0.9)*
Race		
Chinese	1.0	1.0
Malay	1.2 (1.0, 1.5)	1.1 (0.9, 1.3)
Indian	1.0 (0.8, 1.3)	1.0 (0.8, 1.2)
Others	1.2 (0.8, 1.7)	0.7 (0.5, 1.0)
Educational level		
PSLE^#^/“O-”/“N-” Levels	1.0	1.0
“A-” Levels/Diploma	1.4 (1.2, 1.7)*	1.3 (1.1, 1.5)*
Degree	1.4 (1.2, 1.8)*	1.3 (1.1, 1.6)*
Occupational status		
Employed	1.0	1.0
Unemployed/retired	0.7 (0.6, 0.8)*	0.8 (0.7, 0.9)*

^#^PSLE: Primary School Leaving Examination; *P < 0.05; ^a^OR: odds ratio; ^b^CI: confidence intervals.
